# Solid-State Fermentation Towards Sustainability and Circularity in the Bioprocessing of Agri-Food Industrial Wastes

**DOI:** 10.3390/foods15091482

**Published:** 2026-04-24

**Authors:** Carlos N. Cano-González, Eliseo Cárdenas-Hernández, María de la Luz Herrera-Estrada, Miguel Angel Aguilar Gonzalez, José L. Martínez-Hernández, José Sandoval-Cortes, Cristóbal N. Aguilar

**Affiliations:** 1 Bioprocesses and Bioproducts Research Group, Food Research Department, School of Chemistry, Universidad Autónoma de Coahuila, Saltillo 25280, Coahuila, Mexico; ccanogonzalez@uadec.edu.mx (C.N.C.-G.); eliseo.hernandez@uadec.edu.mx (E.C.-H.); luz.herrera@uadec.edu.mx (M.d.l.L.H.-E.); jose-martinez@uadec.edu.mx (J.L.M.-H.); josesandoval@uadec.edu.mx (J.S.-C.); 2 Cinvestav Unidad Saltillo, Parque Industrial Ramos Arizpe, Ramos Arizpe 25903, Coahuila, Mexico; miguel.aguilar@cinvestav.edu.mx

**Keywords:** agri-food waste, biocompounds, bioprocesses, biotechnology, circularity, sustainability, zero-waste goal

## Abstract

Solid-state fermentation (SSF) is a pivotal biotechnology in the circular economy, leveraging agri-food industrial waste and byproducts to produce high-value bioproducts while minimizing organic waste. By aligning with sustainability goals and zero-waste principles, SSF enables the production of enzymes, bioactive compounds, and secondary metabolites for food, agriculture, and biomedical applications. Recent advancements have optimized critical parameters, including substrate selection, culture conditions, and scalable bioreactor designs, enhancing process efficiency and reducing environmental impact. Despite progress, challenges persist in maximizing production yields and fostering industrial adoption. Addressing these hurdles, particularly through integrated environmental and techno-economic analyses, is essential to solidify SSF’s role as a sustainable and competitive bioprocessing method. This review analyzes the latest advances in SSF, including the valorization of food and agro-industrial wastes, innovative bioreactor designs, microbial engineering for more efficient strains, bioenergy production and its integration into biorefineries, and contributions to the circular bioeconomy. Thus, SSF emerges as a key technology in sustainable industrial biotechnology, offering eco-friendly alternatives and promoting a more efficient production model.

## 1. Introduction

**Figure 1:**
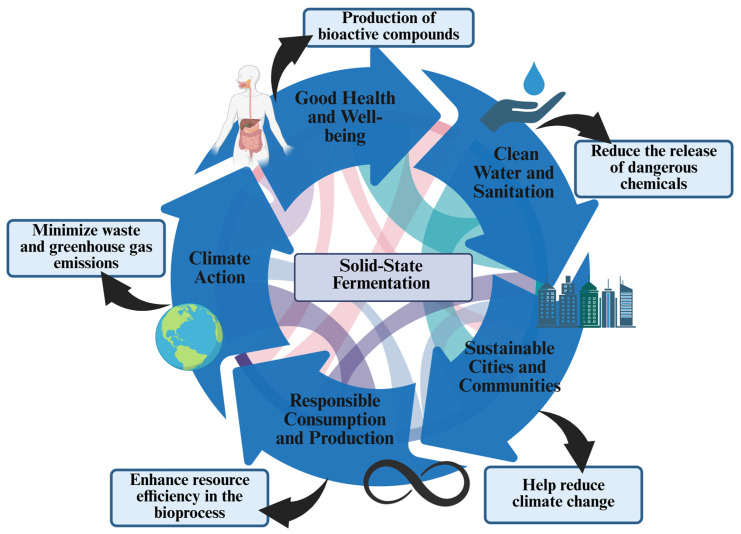
SSF as a global zero-waste strategy and its contributions to sustainability.

The world is undergoing rapid human-driven environmental change. The United Nations established the Sustainable Development Goals (SDGs), which include an action plan to ensure prosperity and symbiotic partnerships to sustain biodiversity [1]. An important strategy is global zero-waste, which focuses on using discarded materials as a resource to reduce waste accumulation and produce value-added products. The goal of zero waste is to stimulate sustainable production and consumption, achieving a circular economy [1,2,3]. This strategy encompasses the global goals of Good Health and Well-Being, Clean Water and Sanitation, Sustainable Cities and Communities, Responsible Consumption and Production, and Climate Action (Figure 1) [4]. The circular economy and sustainable environmental practices represent an innovative, rehabilitative approach that emphasizes conserving natural resources and minimizing waste while moving away from the linear model [5]. The circular bioeconomy prioritizes waste valorization for the production of value-added biocompounds and the development of technologies. The efficient valorization of waste offers a more sustainable approach, generating high-quality, marketable products with physical, chemical, and biological properties to meet one or more needs [6,7]. The circular economy can be used in conjunction with biotechnology to boost local businesses, protect the environment, and develop strategies such as the bioprocessing of agri-food industrial waste and byproducts to generate value-added compounds [8].

The increase in waste over recent decades is globally significant. Organic waste from food, agricultural, industrial, and domestic sources has caused environmental problems, and the solutions implemented are insufficient. The food processing industry generates over 1.3 billion tons of waste annually [9,10]. It is, therefore, important to consider innovative alternatives for efficient waste management. Given the variable composition of waste, it can be used to obtain value-added compounds. These compounds can be extracted from waste or produced as value-added compounds by microorganisms [9,11,12]. Another recent approach is the generation of bioenergy (bioethanol, biodiesel, biohydrogen, and biomethane) through anaerobic metabolism [7]. The valorization of agri-food waste and byproducts is important to generate value-added products, including biopolymers (carbohydrates, proteins, fats), enzymes, phenolic compounds, pigments, biosurfactants, biostimulants, and biofertilizers. It is essential to assess the sustainability of the process through a life cycle assessment, accounting for its environmental impacts and benefits relative to existing industrial methods. Similarly, the process’s profitability should be evaluated to determine the cost–benefit ratio [13]. The process can potentially drive innovation and technological advances in waste management [6,13].

The main requirements for processes to obtain biocompounds, whether for research or industry, are profitability, simplicity, and efficiency. Therefore, solid-state fermentation (SSF), which applies microorganisms—mainly bacteria, fungi, and yeast—on waste that acts as a wet solid substrate under aerobic conditions, is a sustainable way to develop value-added compounds [6,7,11,14]. A prominent approach is the utilization of microbial communities to degrade engineered materials. Microorganisms, particularly extremophiles, will play a major global role as technological drivers during this shift. Their genomes hold the genetic blueprints for sustainable biotechnology [15]. Thermophilic microorganisms will represent a transition that will include active, future-oriented fundamental and industrial research [8,15].

One of the critical points for scaling up the process is the choice of reactor design, which is influenced by the heat transfer capacity of the selected waste substrate, thus mitigating potential thermal problems [7]. This approach is based on the dynamics of microbial growth, adaptation mechanisms, and metabolic activity, which are fundamental to the system’s performance [9,11].

Fermentation is increasingly adopting sustainable practices, which impacts the local production of genetically modified foods, mainly meat, milk, and egg protein substitutes [16,17]. This type of fermentation is marked by the development of functional ingredients, the replacement of animal-derived inputs, and the production of nutraceuticals and flavor compounds (proteins, enzymes, and bioactive compounds) with high efficiency and purity [16]. Metabolic engineering and synthetic biology are tools that greatly influence the precision fermentation process by genetically modifying microorganisms to optimize metabolic pathways, boost product yield and quality, and enhance process stability [18,19]. This fermentation type helps reproduce existing market products or create trend-driven innovations to meet population needs through sustainable practices [20]. Globally, the pharmaceutical, cosmetic, food, chemical, and energy industries are the main beneficiaries of microbial biotechnology-based solid-state fermentation (SSF) applications [21]. This review highlights the essential role of solid-state fermentation (SSF) in the circular economy. Its main goal is to identify the key areas where SSF, as a bioprocess, supports the zero-waste strategy. The review emphasizes optimal conditions and substrate selection for producing compounds that reduce organic waste (food and agro-industrial) and enhance resource efficiency. It also explores the valorization of waste to create bioactive compounds and bioenergy. Additionally, SSF emerges as a vital process that fosters sustainable practices and advances a circular economy. This process has diverse applications and stimulates research and innovation in biotechnology.

## 2. Fundamentals of Solid-State Fermentation

Microorganisms can produce secondary metabolites [22]—complex biological compounds that are not involved in growth or reproduction but have other important functions after the growth phase [23]—through SSF. These compounds have industrial applications in the development of pharmaceutical, food, and cosmetic products [23,24]. Most of the microorganisms used in SSF, such as fungi and yeast, are considered safe, as toxin-free secondary metabolites are obtained, allowing their use in humans [25]. The SSF bioprocess is very important because it allows for the production of compounds that cannot be synthesized by submerged fermentation (SmF) [23].

SSF offers many advantages: low energy consumption, prolonged product stability, reduced or eliminated organic wastewater discharge, and affordable production costs [23,25]. Utilizing microorganisms in bioprocesses will accelerate the shift towards a circular and carbon-neutral bioeconomy. This area will necessitate interdisciplinary foundational and industrial research, focusing on applied biotechnology and process optimization to guarantee scalability and sustainability [26]. However, the full potential of SSF has not been demonstrated at a commercial scale [25], underscoring the importance of improving SSF processes.

### 2.1. SSF Processes and Factors

SSF is a process where microorganisms, such as bacteria, fungi, and yeast, are cultivated in a moist, solid, insoluble organic material that acts as a support and source of nutrients, allowing the microorganisms to grow in the absence or near absence of free water [25,27], similar to their natural environment (mainly for fungi), which makes the process less susceptible to microbial contamination and allows for higher enzymatic efficiency [24]. However, for compounds to be produced, the microorganism must be exposed to stress conditions, such as the deficiency in an essential nutrient; for example, in *Penicillium chrysogenum*, the deficiency of glucose in the medium allows the consumption of lactose, which induces the biosynthesis of penicillin [23].

**Figure 2:**
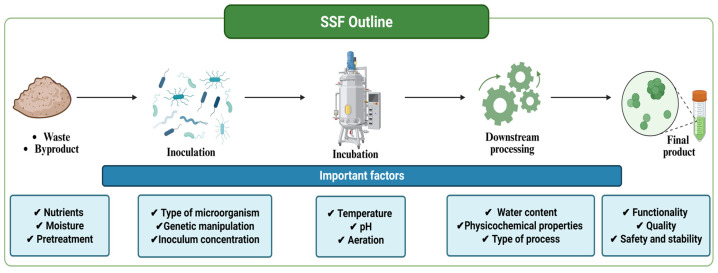
Diagram with the key factors in the SSF.

SSF involves a series of steps: first, the preparation of substrates and growth media, as well as the isolation of the microorganism used [28]. The microorganism is then inoculated into the substrate, initiating fermentation. During this process, the final products are obtained, i.e., primary and secondary metabolites [25]. This SSF process is carried out on substrates with low moisture content, which offers advantages for the growth of the microorganisms [22]. Through the mycelium, fungi penetrate the substrate to biotransform it, while bacteria and yeast act on the substrate surface [22,23]. The carbon in the substrate is converted into CO_2_ and biomass, which decreases the growth of microorganisms; CO_2_ released and O_2_ consumed must be constant for the correct growth of biomass and secondary metabolites (Figure 2) [22,26]. Additionally, the production of primary and secondary metabolites is controlled by numerous factors, such as the type of microorganism, substrate composition, temperature, light, pH, aeration, agitation, sterilization, and reactor type [23,24,27]. These factors contribute to understanding fermentation systems by elucidating gene expression, enzyme activity, microbial community composition, and metabolite dynamics. Metabolomic and genomic knowledge enables predictive mechanistic modeling and rational optimization of fermentations across different biotic and abiotic factors. This is achieved by providing information on how microbial ecology and metabolic pathways respond to variations in strain selection, substrate composition, or fermentation conditions [24,27].

### 2.2. Microorganism Used in SSF

The choice of microorganism for SSF depends on many considerations, such as the microorganism’s growth behavior, product yield, ability to produce the metabolite of interest, ability to degrade the substrate, tolerance to fermentation conditions, and product safety [25]. Some bibliometric analyses have shown that fungi are the most commonly used microorganisms for SSF, mainly those of the genus *Aspergillus* [25]. However, bacteria and yeast are also used [23]. In general, fungi comprise the genera *Trichoderma*, *Aspergillus*, *Penicillium*, *Fusarium*, and *Rhizopus*, and edible fungi have also been applied in SSF [26,29]. Bacteria such as *Bacillus mycoides*, *Bacillus megaterium*, and *Lactobacillus* spp.—which include *L. plantarum*, *L. bulgaricus*, *L. acidophilus*, *L. delbrueckii*, *L. coryniformis*, and *L. rhamnosus* [25]—have been studied. However, the range of products that can be developed is limited when using bacteria, for example, in enzyme production [26]. Yeasts such as *Saccharomyces cerevisiae*, *Saccharomyces boulardii*, and *Candida* sp. are more common in the production of biofuels and extracellular proteins [25,26]. Actinobacterial species (*Streptomyces chattanoogensis* and *Streptomyces thermonitrificans*) have also been used in SSF [25]. Yeast and filamentous fungi can utilize solid organic substrates with low moisture content, primarily for the production of biofuels, enzymes, and primary and secondary metabolites. Gram-positive bacteria can efficiently colonize such substrates, produce numerous enzymes and metabolites, and tolerate adverse environmental conditions. Bacteria have simpler genomes and reproductive and growth mechanisms than eukaryotic microorganisms [25,26].

### 2.3. Inoculum Size and Type

Inoculum size refers to the amount of the microorganism; for example, in the case of fungi, it refers to the amount of spores or mycelium that is added to the substrate and affects the growth rate and biomass production [26]. A low concentration of inoculum may not be adequate for the initiation of microorganism growth, and a high concentration leads to problems, such as limitations in mass transfer [24]. In SSF, inoculation is initiated with a mycelial or spore inoculum. Generally, spores are the most commonly used type of inoculum, but some studies have shown that certain microorganisms, such as *Chaetomium cellulolyticum*, must be inoculated in mycelial form to generate a greater amount of metabolites [23].

Substrate characteristics can affect the optimal inoculum size for a particular SSF process. If the substrate is very porous, a larger inoculum size may be necessary to ensure that the entire substrate is colonized [28,29,30]. However, a smaller inoculum size may be sufficient if the substrate is less porous. By monitoring fermentation progress, the inoculum size can be increased or decreased as necessary to optimize the yield and quality of the final product [26]. Another important factor is inoculum density, as a higher density minimizes contamination with unwanted organisms [23]. Inoculum concentration varies widely depending on the microorganism used in SSF processes, but there are optimal ranges for effective inoculation. For filamentous fungi, a range of 1.0 × 10^7^ to 4.0 × 10^7^ spores or colony-forming units (CFUs) per gram of solid substrate is recommended. For yeast, optimal concentrations range from 1.5 × 10^7^ to 1.0 × 10^8^ viable cells/g of substrate, while for bacteria, the typical inoculation represents 1% to 25% (*v*/*w*) or a range of 1.0 × 10^6^ to 1.0 × 10^8^ CFU/g of the total mass of the solid medium [26,30].

### 2.4. Substrate

Substrates for SSF have relatively low moisture content (<50%) but are suitable for microbial growth [23,31]. In addition, they provide all the necessary nutrients for growth and metabolism [26]. The substrates in SSF serve as a source of nutrients and as a support material for microorganism growth [22,23,26]. Synthetic materials that serve as support include minerals (vermiculite and perlite), polymeric resins (Amberlite), polystyrene, polyurethane foam (PUF), and silica gel, which are supplemented with nutrients (sources of carbon, nitrogen, vitamins, and minerals). Natural materials include agri-food wastes and byproducts such as those generated by the cereal industry (wheat bran, rice, barley, and corn bagasse) [22,26], the fruit and vegetable industry (pulps, peels, and seeds, as well as stems, leaves, and roots) [27], the sugarcane industry (bagasse), the coffee industry (coffee pulp), the oilseed industry, and the wood industry, among others [32]. These materials are rich in sugars, starch, lignin, cellulose, and polysaccharides, which effectively provide nutrients [24,26]. Natural materials have the advantage of being usable in greater quantities and of converting waste or byproducts into value-added products, enabling a more comprehensive approach to utilization, compared to synthetic materials, which serve primarily as supports and do not incorporate waste as effectively.

Substrate selection is important because the growth and physiology of the microorganism are influenced by the solid support [23]. However, the most important factor is cost; therefore, agri-food industrial wastes and byproducts are preferred, but differences in their physicochemical characteristics affect the production of compounds [33]. Despite this, the main advantage is the operational ease and the similarity to the natural habitat in which microorganisms grow and develop; consequently, research on agri-food industrial wastes and byproducts as substrates for SSF has increased [23,24]. Additionally, waste disposal is a concerning and widespread problem related to rapid population growth and urbanization, coupled with poorly integrated management strategies; thus, SSF has emerged as an innovative tool for the valorization of these materials and their conversion into value-added products [24,33].

### 2.5. Pretreatment of the Substrate

Agro-food-industrial wastes and byproduct transformation processes require efficient pretreatment steps to release compounds of interest. Pretreatment is important because it improves nutrient availability and makes them more accessible to microorganisms, primarily by increasing the substrate surface area [34]. Pretreatment includes particle size reduction by grinding, as well as physical, chemical, or enzymatic hydrolysis, which helps improve substrate accessibility [24]. Among the available strategies, enzymatic and chemical pretreatments have proven particularly versatile for depolymerizing complex biopolymers. These methods present complementary characteristics that make them suitable for different raw materials and industrial applications [12,34]. Enzymatic systems stand out for their extraordinary specificity of action, enabling the controlled hydrolysis of complex substrates such as cellulose, hemicellulose, and proteins. This group of biocatalysts, which includes cellulases, amylases, pectinases, proteases, lipases, and ligninases, acts through precise molecular mechanisms. The main advantage of this approach lies in its selectivity, which minimizes the formation of undesirable byproducts and reduces the energy requirements of the process [35,36]. The choice of methods must balance technical and economic aspects. While enzymatic systems offer greater process control and lower environmental impact, their cost remains a significant limitation for industrial-scale applications [35,36]. Chemical pretreatments offer robust solutions for the large-scale processing of lignocellulosic materials. Their efficiency critically depends on three factors: the chemical nature of the substrate, operating conditions (pH, temperature, time), and subsequent detoxification protocols. Acid treatments, which commonly use HCl, H_2_SO_4_, or HNO_3_, are particularly effective for hydrolyzing hemicellulosic components. In contrast, alkaline treatments with NaOH or Ca(OH)_2_ exhibit a greater affinity for the lignin fraction, thereby improving the accessibility of polysaccharides to subsequent enzymatic action [36]. Chemical treatments, although more economical in terms of reagents, require specialized equipment and generate waste streams that must be properly managed [10,36]. Another approach to prepare the substrate is to use other microorganisms to break it down before fermentation [37].

Particle size is important because it affects particle surface area and volume, which in turn influence the accessibility of microorganisms and packing density [23,38]. In addition, a correct size allows for efficient gas exchange and good mass and heat transfer [24]. Particle size determines the void space occupied by air, thus influencing oxygen transfer and affecting growth. Adequate particle size increases mass transfer [24,38]. The smaller the size, the greater the surface area for microorganism growth, but particles that are too small can agglomerate, leading to substrate agglomeration, thus interfering with microbial aeration. While a larger size provides better aeration, the surface area available for growth becomes limited [9,39]. Therefore, the appropriate size will depend on the substrate and the microorganism. As a result, many works on culture optimization focus primarily on particle size. Some studies that highlighted particle size included the following: in the production of enzymes from barley husks, a particle size of 1.0 mm was used [40]. In the case of bioactive compounds, apple and agave mezcal were used, with a particle size of 0.841 mm [41], while in another study, Mexican rambutan peel was used with a particle size of 2 mm [42].

### 2.6. Nutrients

When synthetic supports or agri-food industrial wastes and byproducts with a low nutritional level are used, it is necessary to incorporate nutrients into the fermentation medium, mainly because they allow for the proper growth of the microorganisms [22,24]. Nutrients are classified based on the parameters of fermentation cultures, including carbon and nitrogen sources, minerals, and other essential compounds [22,24,31]. Carbon serves as a source of energy, and molecules such as glucose, cellulose, starch, sucrose, and lactose serve as examples. Nitrogen sources include amino acids, peptones, and urea. Organic acids, vitamins, growth hormones, and minerals such as sodium, nickel, copper, manganese, potassium, iron, calcium, zinc, magnesium, and molybdenum are also incorporated [31]. Certain elements, such as phosphorus, sulfur, and metals, although present in limited amounts, are also essential [24].

### 2.7. pH

pH is one of the most important parameters, as it affects the metabolism and growth of microorganisms [24]. Furthermore, in SSF, the type of substrate and lack of free water make it difficult to monitor pH changes, although the type of substrate may have a buffering effect due to its chemical complexity [23,43]. Additionally, the secretion of organic acids causes pH changes [24]. Bacterial contamination can be prevented in fungi and yeast by growing at a pH different from that suitable for bacteria, as fungi and yeast have a wide pH range for growth that can be exploited [44]. The pH range for bacteria is 4 to 8, but it can vary due to acidophilic bacteria. Filamentous fungi grow at pH 2–9 (3.8 to 6.0 is the optimum range), while yeast grows at pH 4–5 (2.5 to 8.5 is the optimum range) [24]. Ammonium salts have been used in combination with urea or nitrate salts in SSF to neutralize the effects of acidification and alkalinization [23]. Interestingly, previous studies have shown that several fungal species, such as *Aspergillus niger* and *Rhizopus oryzae*, can significantly reduce substrate pH during fermentation [26]. pH has a direct relationship with microorganism activity, which in turn affects fermentation rate and product yield. This parameter is influenced by the production of organic acids and CO_2_; therefore, pH control during fermentation is crucial for maintaining production yield [23,43].

### 2.8. Moisture Content and Water Activity (Aw)

Optimal moisture content is required for microbial growth, as low moisture content reduces the solubility of substrate nutrients, while high moisture content reduces the porosity of the solid matrix, affecting oxygen transfer and limiting enzymatic performance [44,45]. In addition, microbial growth and evaporation reduce substrate moisture, leading to dryness and affecting the SSF process [38]. SSF moisture levels range from 30 to 85%. The solid matrix should contain at least 70% moisture for bacterial growth and 20–70% for filamentous fungi [46]. On the other hand, Aw, the water available for microbial growth, allows for the identification of the most suitable microorganisms for the SSF process. Because SSF develops in a limited amount of free water, bacteria—having a high Aw requirement (0.8–0.9)—are less effective than fungi and yeast (0.5–0.6) for SSF [23,26]. This means that microorganisms adapted to low water activity levels can thrive better in SSF processes [24]. In addition, low Aw can affect the growth of microorganisms and delay metabolite production [47,48].

Moisture content is important because it allows for lower wastewater levels. In addition, the incorporation of agri-food industrial wastes and byproducts provides a high moisture content, so the addition of water to the substrate is not required, although a light drying process might be necessary [24,49]. Although they are parameters related to the presence of water in a matrix, humidity indicates the amount of water present, while Aw indicates the fraction of water available for microorganisms and reactions [24,49].

### 2.9. Temperature

The optimal temperature for microorganism growth and primary and secondary metabolite production depends on the type of microorganism and the bioreactor; fungi have a broader growth temperature range than other microorganisms [23,50]. However, the temperature inside the bioreactor increases with growth rate and metabolic activity; consequently, it is difficult to control. Although aeration and agitation can help regulate the temperature, they have even been shown to introduce a large amount of air through a gaseous vent to avoid overheating and subsequent condensation, which increases the amount of water in the matrix and negatively affects the growth of the microorganism [23,26,43].

If the temperature is too low, growth may slow or stop, resulting in decreased substrate utilization and metabolite production [26]. On the other hand, if the temperature is too high, microorganisms may die, or toxins may be produced, resulting in an unsafe and undesirable fermentation product [26,51]. However, identifying the optimal temperature for efficient production of a specific product is essential. Temperature limits for each microorganism are critical, especially when the upper threshold is exceeded, which is more common due to heat transfer in a bioreactor. If the production of a compound is not controlled, the yield can decrease significantly or lead to the formation of unwanted byproducts [26,47].

### 2.10. Other Indispensable Factors

There is undoubtedly a wide variety of factors, some more important than others. In general, key factors that must also be considered include sterilization, aeration, and agitation. The sterilization process, which eliminates infection-causing microorganisms, keeps the culture pure and safe [23,24]. Aeration should be considered in SSF because it affects oxygen transfer, heat removal, and homogeneous nutrient distribution. Aeration regulates oxygen transfer and the removal of CO_2_ and heat. Agitation helps to avoid substrate compaction and microorganism damage [24,26]. Because these factors are of paramount importance, engineering has rapidly developed bioreactors to overcome scaling-up challenges and to monitor critical factors in real time, including heat and mass transfer [24,26].

### 2.11. Products Obtained from SSF

After the SSF process, it is necessary to recover the compounds through extraction of the fermented matter, either by mechanical extraction or with the use of solvents [52,53]. At this point, factors such as the type of solvent and its concentration, the solid/liquid ratio, and pH have a significant effect [23]. Once the compounds are obtained, they must undergo purification processes. In addition, the fermented solid can have applications in animal feed or biogas generation [23,24]. The products obtained vary depending on the metabolite of interest, the substrate and the microorganism, but in general, antibiotics, bioactive compounds, phenolic compounds, enzymes (amylases, cellulases, xylanases, proteases, phytases, lipases, galactosidases, pectinases, and ligninases), organic acids, xanthan gum, unicellular protein, and antioxidants, among many others, can be obtained by SSF [23,51,52,54]. All these compounds exhibit functional properties that impact applications in the food, pharmaceutical, and agricultural sectors. Due to the need for functional products, SSF has served as an alternative for their production. Table 1 lists various microorganisms that use different substrates to produce products with industrial applications.

## 3. Innovation Perspectives in the Solid-State Fermentation Process

Microbial production of bioproducts has long been pivotal in the food, pharmaceutical, and biotech industries [25]. In recent decades, SSF has gained significant scientific attention due to its ability to valorize agro-industrial waste and enhance the synthesis of high-value compounds [23]. Its industrial adoption continues to grow, driven by advantages in sustainability, cost reduction, and process efficiency [21,22,23,24]. This makes it highly competitive compared to other biotechnological processes, such as SmF. SSF is highly competitive in sectors where production costs and sustainability are key factors, promoting the circular economy [65,66,67]. However, large-scale implementation faces technological challenges in controlling temperature, humidity, and oxygen, which limit its large-scale application [23,46]. Combining SSF with advanced biotechnology strategies, such as synthetic biology and automation, can improve its competitiveness in the future. The arrival of biotechnological innovations, mainly regarding bioproducts obtained through fermentation and bioprocessing, has opened many new avenues for the application of SSF [68].

**Figure 3:**
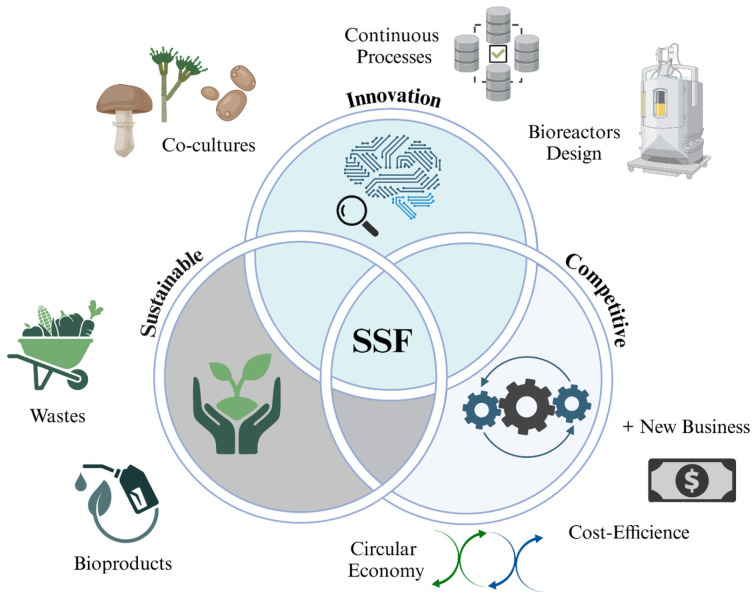
Environmental and innovation perspectives in solid-state fermentation.

Constant updating and innovation of a process are fundamental to fully realize its potential over time and maintain its wide use. Innovation enables the improvement and optimization of various aspects, such as efficiency, sustainability, and competitiveness, in a constantly evolving environment. Achieving adaptability to new demands and cost reduction drives research and development to improve process performance, opening new opportunities for product development and the implementation of more efficient, sustainable, and competitive technologies [69,70]. From an environmental perspective, innovation in bioprocesses significantly reduces ecological impact, promotes the use of renewable materials, and minimizes waste generation (Figure 3) [21,23]. In SSF processes, innovation has been developed in areas such as co-culture use, genetic improvement, substrate optimization, continuous processes, and the design of more efficient bioreactors [46,71,72,73]. These innovations have allowed SSF to evolve from a traditional technology to an advanced tool for producing high-value metabolites across various industries [74].

### 3.1. Strategies in SSF

The use of co-cultures in SSF processes is an innovative strategy that consists of combining two or more microorganisms to improve process efficiency and the production of high-value metabolites [75]. Natural microbial interactions inspire this technique and have proven to be highly beneficial in terms of yield, product diversity, and the stability of the fermentation system [76]. Understanding the biological interactions that modulate the association between microorganisms enables the development of specific co-cultures or consortia, whether synthetic or natural. The ability to accurately predict and manipulate cellular interactions is a key element in advancing synthetic biology. To implement the use of co-cultures, it is necessary to ensure that the microorganisms involved can survive under the same conditions [76,77]. In addition, the use of co-cultures enables greater process stability and a broader diversity of desired products. Recent studies have utilized combinations of lactic acid bacteria, filamentous fungi, and yeast, as well as ascomycetes and basidiomycetes [23,76,77,78]. Among the benefits of implementing co-cultures is greater production of metabolites of interest due to synergistic interactions between microorganisms and the combination of their different metabolic pathways, which optimize the conversion of substrates into the products of interest [76]. Some disadvantages could include antagonism between microorganisms, inhibition of microbial metabolism, and, in some cases, binding reactions between irrelevant metabolites that affect production yield. Furthermore, fermentation conditions can negatively impact some microorganisms if an equilibrium is not reached [76,77].

The use of continuous bioprocesses in SSF enables sustained production of metabolites, enzymes, and other biotechnological products by maintaining a constant flow of substrate and microorganisms. This strategy offers advantages such as increased productivity, reduced costs, optimized substrate use, and lower waste generation, making it more sustainable and efficient than batch processes [21,74,75]. For its implementation, fixed-bed or percolated-bed reactors, continuous feeding and extraction systems, microorganism immobilization, and inert supports that promote culture stability are used. However, these processes have disadvantages, including limited control over environmental parameters (temperature, humidity, oxygenation), accumulation of inhibitory metabolites, potential contamination by unwanted microorganisms, and difficulties with automation and industrial scaling [76]. Despite these challenges, it remains a process that stands out for its relevance in modern biotechnology across the pharmaceutical, food, and agricultural industries, thanks to its efficiency and low environmental impact [58].

### 3.2. Design and Control of Bioreactors

Solid-state fermentation presents challenges concerning temperature and humidity control. In this technique, the substrate contains a limited amount of water that can generate large variations in moisture percentage [6]. Due to small water losses through evaporation, the temperature can also vary considerably from the ideal temperature for fermenting microorganisms, particularly at the base of the fermenters and on the surface of the substrate [16]. Hence, studies dedicated solely to the evaluation of these two parameters must be carried out, as in the case of protease production by *Aspergillus uvarum*. This study showed that maintaining adequate humidity and temperature allows for maximized protease production and scaling up the process [77].

Another challenging aspect of SSF bioprocess design is the physicochemical characteristics of the substrate, such as granulometry and the compressibility index [5]. It has been shown that these parameters play a determining role in heat transfer phenomena, maintaining the temperature during fermentation throughout the reactor, regardless of the geometry, since, with high granulometries and low compressibility indices, the gas flow through the substrate is adequate [78], which also allows for good oxygenation and CO_2_ elimination. This remains a challenge in SSF but is achieved effectively even in tubular reactors [79]. Therefore, for proper control of SSF processes, in addition to the parameters mentioned above, it is recommended to implement advanced sensors and monitoring systems that enable real-time adjustment of conditions, as well as the design of more efficient bioreactors for large-scale production.

Because laboratory and pilot scale studies have shown that SSF has certain advantages over the submerged fermentation technique (SmF), efforts and resources have been invested in research to take advantage of its true potential, given that it has faced some limitations for industrial-scale implementation [78]. The main cause is the lack of efficient and scalable bioreactor designs, which must be supported by mathematical models and automated control systems. It is important to efficiently maintain heterogeneity in temperature and mass and operate the process aseptically, as maintaining sterility is a major challenge [71,78]. Thus, there is still significant scope for research into the development of SSF bioreactors, thereby facilitating the process and its applications in the biotechnology industry. According to their mode of operation, bioreactors are classified into four categories based on their design characteristics, the impact of operating conditions on productivity, applications, and limitations [71]. Over the years, specific modeling studies have been reviewed and presented to address these limitations and thus enable the implementation of the SSF process on a larger scale and optimize the processes [62,79]. In the future, SSF is expected to reach a level of development comparable to SmF, provided rationalization, innovation, and process standardization continue in line with current trends.

The types of reactors used for SSF are bag, tray, stirred-drum, and packed-bed. The type of reactor depends on the heat transfer capacity of the selected waste, but porosity and effective aeration can facilitate heat transfer, minimizing the problem. In addition, control over parameters such as temperature, humidity, and oxygen enables effective scale-up [7]. Bag reactors are economical but have a limited scale and are susceptible to contamination. Tray reactors promote oxygenation but require rigorous control of conditions. Stirred-drum reactors offer excellent homogenization, although they have high energy consumption and maintenance costs. Packed-bed reactors, on the other hand, optimize mass transfer but can present clogging and distribution problems. The choice of reactor depends on the production scale and process conditions [9,11]. The implementation of sequential batch reactors is among the operational strategies to improve efficiency during scaling up. The impact of this strategy is to reduce time and costs by using fermented material as an inoculum in subsequent SSF cycles [7,78]. SSF comprises three stages: upstream, midstream, and downstream. The upstream process focuses on valorizing waste to produce the compound of interest for extraction, including optimization of carbon and nitrogen sources and inducers for microbial production of desirable compounds. This process involves the growth, adaptation, and metabolism of microorganisms [9,11]. SSF also has as its main focus environmental bioremediation because it is a versatile approach for different contaminating agents and is widely used at the same site, minimizing the need for transportation and the use of harmful agents [9]. The role of the bioreactor is to provide a controlled environment for the metabolism and growth of microorganisms [26,79]. Several basic bioreactors have been analyzed for SSF operation (SSF tray fermenters, SSF rotating-bed fermenters, continuous stirred-tank reactors (CSTRs), and piston-flow reactors (PFRs)) [23]. Tray bioreactors are the most commonly used traditional bioreactors, with a simple design and ease of use for SSF scale-up [24].

### 3.3. Cost Efficiency of SSF

The cost-effectiveness of SSF depends on a comprehensive approach that spans selecting economical substrates, optimizing process conditions, improving product extraction, and managing byproducts [21,46]. Continuous analysis of these areas, along with investments in technologies that enable process automation and scalability, significantly improves the profitability of SSF processes. Since the SSF process is recognized as an efficient, cost-effective, and promising technology that enables microorganisms to grow on the surfaces of a wide variety of solid materials in the absence of free water, it allows for the use of waste from various industries, mainly organic waste [72]. This, in turn, offers the potential for significant cost reductions in substrate processing and decreased contamination of soils and the environment from biomass. These wastes can serve as a rich source of various compounds for the growth of microorganisms and for the production of many goods, including biogas, biofuels, antioxidants, and enzymes, among other valuable compounds mentioned in previous sections [80,81]. In addition, since SSF mimics the natural growth conditions of many microorganisms, the working conditions are usually low risk. The SSF process stands out among other bioprocesses as a technology aimed at producing compounds that benefit society and as a sustainable, profitable, and low-impact process, as it can use readily available substrates, such as food waste, agricultural waste, and urban waste [24,31]. SSF is characterized by a process in which microorganisms grow with virtually no free water, requiring less energy for sterilization. Furthermore, it is less susceptible to microbial contamination and substrate inhibition, ultimately enabling increased enzyme productivity and a consequent reduction in operating costs, primarily from mixing and heating [82,83]. Few studies address the techno-economic analysis of SSF, but some demonstrate the feasibility of producing enzymes (hydrolases), biopesticides, biofertilizers, and bioenergy. All of this depends on the microorganism, the agri-food industrial waste and byproducts, and the bioreactor design [82,84,85,86]. The techno-economic indicators used to assess profitability under current conditions include net production cost, gross profit margin, investment payback period, return on investment (ROI), and net present value (NPV). The analysis in this study revealed that the most profitable approach is to produce enzymes in situ and valorize the solid fraction via SSF, yielding an internal rate of return of 13%. This approach offered high profit margins (74%) and a 6-year investment payback period. Furthermore, the joint production of higher-value bioproducts and energy reduced the reliance on policy instruments for organic waste treatment while maintaining energy self-sufficiency [87]. Therefore, applying environmental and techno-economic impact analysis methodologies to SSF processes is important to obtain different compounds of interest and compare them with those obtained using traditional methods. This is a challenge, but it contributes to SSF’s sustainability and innovation.

## 4. Challenges of SSF in Sustainability and Circularity

In the last decade, SSF has advanced significantly, consolidating itself as a key biotechnological technique for sustainability and the circular economy. The rapid growth of the global population, coupled with accelerating economic development and urbanization, has significantly increased solid waste generation. This includes diverse types such as agricultural and agro-industrial waste, household waste, and waste from both humans and animals [7,9]. Recent estimates suggest that global municipal solid waste could reach 3.4 billion tons annually by 2050 if current trends continue, highlighting the urgent need for sustainable waste management solutions [10]. Although developments have occurred in different directions, significant advances have focused on specific areas that have had a greater impact on social, economic, and environmental levels. In this way, sustainable development plays an important role in various industries and social sectors while being beneficial to the environment [8].

### 4.1. Zero-Waste Goal: Valorization of Agri-Food Industrial Waste

SSF has been used to transform food and agro-industrial waste into value-added products. For example, it has been used to produce fruit and rose aromas from sugarcane bagasse and beet molasses [88], offering a sustainable alternative for waste management and the production of aromatic compounds. Several authors emphasize the potential of SSF in utilizing agri-food industrial wastes and byproducts like wheat bran, rice husk, and fruit peels as substrates [14]. This not only provides a cost-effective medium for microbial growth but also addresses environmental concerns associated with the disposal of these residues. Through SSF, these substrates can be transformed into value-added products, contributing to waste valorization and sustainability.

A topic of current importance highlights recent advances in the production of lignocellulolytic enzymes by SSF [32] using agri-food industrial waste and byproducts as substrates, enabling the efficient production of cellulases, xylanases, and pectinases. One of the main challenges in this field is the scalability of the process. Although recent research on bioreactors for SSF has been conducted, the design of fully automated systems remains a limitation. The lack of optimized large-scale bioprocesses hinders the industrialization of these technologies [78]. Despite this, SSF remains a promising strategy to valorize agri-food industrial waste and byproducts, promoting the sustainable production of enzymes with applications in biorefineries and the food industry. Several authors analyze various food industry wastes that can be valorized through SSF. The main waste materials used are wheat bran, corn straw, rice husk, sugarcane bagasse, and orange peel to produce enzymes that degrade plant cell walls for the extraction of biocompounds. Waste from the wine and oil industry includes pomace. SSF applied to these wastes can generate enzymes such as laccases and phenolic compounds with antioxidant activity [89].

### 4.2. Solid-State Fermentation in Sustainability

SSF has established itself as a sustainable alternative to traditional submerged fermentation systems, standing out for its lower environmental impact and efficient resource use. This technique, which uses solid substrates with limited moisture, offers key advantages in sustainable industrial production [90]. This approach reduces dependence on virgin raw materials and mitigates environmental problems arising from the accumulation of agri-food industrial waste [84]. From a waste management perspective, SSF generates fewer effluents than other fermentation systems. The resulting solid byproducts have valuable applications as biofertilizers or animal feed supplements, thus maximizing resource utilization [84,86]. This characteristic, combined with the absence of toxic solvents in the processes, makes SSF perfectly aligned with the principles of green chemistry [84].

One of its main attributes lies in its low water and energy consumption. Studies show that SSF reduces water use by 30 to 50% compared to liquid systems, in addition to requiring less energy for sterilization and agitation processes, which translates into a significant decrease in carbon emissions associated with the process [82,86]. According to the World Bank report (2020), global municipal solid waste (MSW) generation from both households and commercial suppliers is estimated to reach approximately 2.01 billion tons annually, equivalent to a per capita carbon footprint of 0.74 kg/day [82]. SSF’s carbon footprint is particularly favorable. Its low energy requirements and ability to sequester carbon in the organic substrates it uses can generate negative carbon balances. Life cycle assessments have consistently confirmed their superiority in terms of environmental indicators compared to conventional bioprocessing methods [82,84].

The efficiency of SSF processes heavily relies on the microbial strains employed. Developing robust strains that can thrive under the specific conditions of SSF, tolerate variations in substrate composition, and produce desired metabolites at high yields is a significant challenge [8]. Advancements in genetic engineering and adaptive evolution techniques offer potential solutions by enabling the development of strains with enhanced capabilities. However, the use of genetically modified organisms (GMOs) introduces additional regulatory and public acceptance challenges that must be carefully managed [18,91].

### 4.3. Substrate Selection and Utilization

The choice of substrate is essential in SSF, as it directly influences microbial growth and product yield. Agri-food industrial wastes and byproducts, such as wheat bran, rice husks, and fruit peels, are commonly employed due to their nutrient richness and abundance [7]. Utilizing these wastes not only reduces environmental waste but also lowers production costs. Pretreatment methods, including mechanical, chemical, or enzymatic processes, are often required to enhance substrate accessibility, but these can introduce additional costs and environmental concerns [6]. Therefore, developing efficient, cost-effective, and eco-friendly pretreatment strategies remains a critical area of research [92,93,94,95].

The waste and byproducts used as substrates for SSF are heterogeneous and can vary in composition, availability, and seasonality, making their consistent and efficient use difficult. Developing pretreatment and conditioning strategies to standardize the substrates is one of the challenges to optimizing their use in SSF [96]. For example, food waste is a valuable substrate for developing SSF-based bioprocesses. However, despite its abundance and low cost, compositional variability is an obstacle to its incorporation into SSF bioprocesses [9]. To address variability in composition, it is suggested that fermenting strains that are viable across substrates with wide ranges of carbon and nitrogen sources be used or that the medium be supplemented to reduce variability [13]. Variability implies not only changes in composition; assuming that the waste does not show great differences in its content of any particular component, variability may be related to the presence of different types of compounds such as proteins, lipids or sugars, among others. The recommendation would be to use consortia of fermenting microorganisms that can use each of the different components of the waste, thus achieving the development of processes with technical, economic and practical viability [97].

### 4.4. Process Optimization and Control

SSF processes are inherently complex due to the solid nature of substrates, which poses challenges for monitoring and controlling critical parameters such as temperature, moisture content, pH, and nutrient distribution [9]. The exothermic nature of microbial metabolism can cause localized temperature increases, potentially inhibiting microbial activity or leading to product degradation. Similarly, uneven moisture distribution can result in suboptimal microbial growth [78]. Advanced sensor technologies and real-time monitoring systems are being explored to address these issues, but their integration into large-scale SSF systems is still in the early stages of development. Innovations in bioreactor design, such as improved aeration and mixing mechanisms, are also being investigated to enhance process control and scale-up [68]. For example, Sentís-Moré et al. [98] studied the optimization of a tray bioreactor design for SSF, focusing on precise control of temperature and relative humidity to ensure optimal environmental conditions during fermentation. Similarly, the importance of maintaining optimal moisture levels for microbial activity has been highlighted, suggesting that advanced bioreactor designs and automated control systems can ensure consistent moisture distribution. Additionally, the SSF technology using various bioreactor designs has been reviewed, examining how operational conditions affect process productivity and highlighting the advantages and disadvantages of each design [99,100].

The solid matrix in SSF presents significant challenges for mass and heat transfer. Efficient oxygen transfer is crucial for aerobic microbial processes, yet the dense nature of solid substrates can impede airflow, leading to anaerobic zones and inconsistent product formation [9]. Heat generated during fermentation can accumulate, especially in large-scale operations, due to inadequate heat dissipation, adversely affecting microbial activity and product stability. Addressing these challenges requires innovative bioreactor designs that facilitate uniform aeration and effective heat removal. Strategies such as forced aeration, substrate agitation, and the incorporation of heat exchangers are being explored to mitigate these limitations [92]. Vauris et al. [38] developed a novel method to assess heat transfer in SSF, highlighting the impact of substrate properties on heat dissipation. Similarly, Casciatori and Thomeo [101] investigated heat transfer in packed-bed systems, emphasizing the importance of airflow in temperature control during SSF.

### 4.5. Scale-Up Challenges and Downstream Processing

Transitioning SSF from the laboratory to the industrial scale involves overcoming several hurdles. Maintaining uniformity in large-scale bioreactors is challenging due to the difficulties in ensuring consistent temperature and moisture levels throughout the substrate mass [11]. The design of scalable bioreactors that can replicate laboratory conditions is complex and often requires significant customization. Moreover, the capital and operational expenses associated with large-scale SSF can be substantial. Collaborative efforts between researchers and industry stakeholders are essential to develop cost-effective, scalable solutions that do not compromise process efficiency or sustainability [8]. For instance, the “scale-up strategies for solid-state fermentation systems” in this article discuss the challenges in scaling up SSF processes, emphasizing the need for tailored bioreactor designs to address issues of heat and mass transfer. Additionally, in “Bioreactors in solid-state fermentation technology: Design, applications and scale-up,” the authors review various bioreactor designs and their scalability, highlighting the complexities involved in maintaining process parameters at an industrial scale [7,102]. The main concerns in industrial fermentations, process improvement, and scale-up are maintaining suitable and uniform reaction conditions, minimizing stress on the microorganisms, and increasing the precision of their metabolism. This is done to maximize production and ensure consistent quality. Additionally, scale-up parameters should be kept as constant as possible to develop appropriate strategies for each product, process, and facility [21].

The fermentation process was developed using *Trichoderma harzianum*, with grass clippings and pruning remains or wood chips as the main substrate and reactor packaging. This work showed excellent results in obtaining a biostimulant for plant growth, specifically indole-3-acetic acid. In addition to the positive results, this study highlights significant challenges in scaling up SSF, including microbial contamination [7]. Studies conducted with *Trichoderma asperellum* at the bioreactor scale produced important enzymes (amylase, cellulases, and lipase) for degradation [103]. In another study, pasta waste was used as a substrate with *Aspergillus awamori* for the production of lactic acid. The recovered lactic acid yield was 55%, with a total ionic concentration of 500 mg/L and an enantiomeric purity of 98.1% for L-lactic acid [104].

The recovery and purification of products from SSF are complex due to the solid nature of the substrate and its lower water activity. Efficient extraction methods that minimize solvent and energy use are needed to ensure the overall sustainability of the process [19]. Innovative approaches, such as supercritical fluid extraction and membrane technologies, are being explored to enhance the efficiency of downstream processing in SSF [90]. For instance, supercritical fluid extraction has been recognized for its efficiency and selectivity in extracting bioactive compounds, offering an environmentally friendly alternative to traditional solvent-based methods. Additionally, membrane filtration techniques have been employed to concentrate biological products, thereby reducing water content and facilitating subsequent purification steps [105,106].

### 4.6. Integration with Sustainability and Circular Bioeconomy

Solid-state fermentation offers a viable pathway for converting organic waste into valuable bioproducts, thereby supporting the principles of a circular bioeconomy [14]. By utilizing low-cost substrates such as agri-food industrial waste and byproducts, SSF not only reduces environmental waste but also contributes to the sustainable production of enzymes, biofuels, and other metabolites [9]. Recent studies have explored the conditions required to produce these products and discussed challenges associated with scaling up SSF processes and downstream processing. For instance, Sánchez et al. [92] studied the potential of SSF in transforming organic waste into high-value products, emphasizing its role in the circular bioeconomy. Similarly, Artola et al. [90] discuss the integration of SSF into biorefineries, focusing on its advantages and main challenges in utilizing organic waste. Additionally, the work by Oiza et al. [68] reviews novel applications of SSF for producing bioproducts from various organic wastes, underscoring the importance of optimizing conditions to maximize product yield and quality. Addressing these challenges is crucial for the successful integration of SSF into existing industrial frameworks and for maximizing resource efficiency.

**Figure 4:**
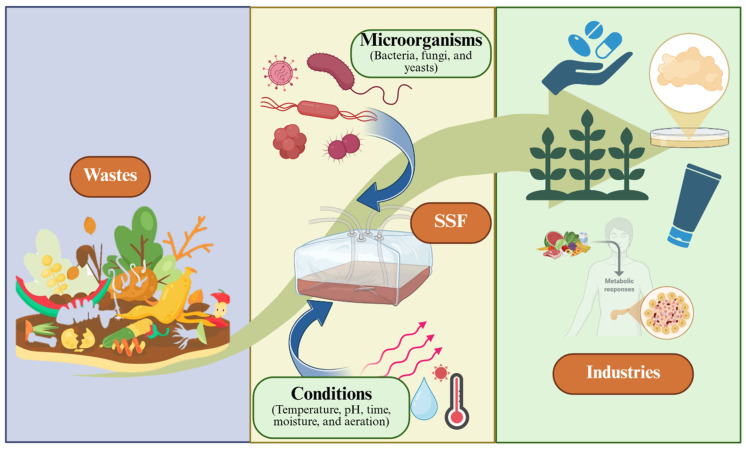
Solid-state fermentation as a strategy for valorization of agro-industrial waste and the production of sustainable bioproducts for industries.

The circular economy is a production and consumption model that seeks to reduce waste sent for final disposal in landfills or confinement centers. It also aims to minimize waste by maximizing the use of raw materials and promoting the reuse, recycling, and regeneration of materials to reduce environmental impact [107,108]. SSF has great potential in the circular economy, as it allows the valorization of agro-industrial waste and the production of sustainable bioproducts (Figure 4) [14,108,109,110]. Circularity measurement uses tools such as the circular economy index (CEI), which measures resource efficiency and waste reduction. Life cycle analysis (LCA) assesses the environmental impacts of the process. Material flow analysis (MFA) calculates the flow and stock of materials. Indicators include the percentage reduction in toxic waste generation and water consumption. This contributes to a more sustainable future [2]. However, SSF faces several key challenges that limit its large-scale implementation.

Finally, technological research and development projects have overcome the above challenges. However, they still face the challenge of achieving social acceptance so that end consumers demand these products in their daily activities; in this regard, the use of waste aligns perfectly with the circular economy concept. This model has been shown to encompass the three pillars of sustainability: the economy, the environment, and social development [7,8]. Decent work, economic growth, and responsible production and consumption can be used in media campaigns to achieve social acceptance [111]. On the other hand, evidence already suggests that we are going in the right direction toward achieving acceptance of bioproducts derived from processes that include the microbiome. This is indicated by the worldwide commercialization of this type of product, ranging from skin treatments to foods with prebiotics and probiotics; this market was projected to reach a value of $1.5 billion in 2025 [112].

While SSF offers a pathway to valorize waste and produce high-value products, its environmental and economic sustainability must be critically assessed. Life cycle assessments (LCAs) are essential for evaluating the ecological impacts of SSF processes, including resource use, greenhouse gas emissions, and energy consumption. Brancoli et al. [83] integrated environmental considerations into the development of an SSF process using surplus bread, identifying inoculum production and the fermentation process as environmental hotspots. Economically, SSF’s viability depends on factors such as substrate availability, market demand for end products, and costs associated with process optimization and scale-up. Developing integrated biorefinery models that combine SSF with other processes can enhance both environmental and economic sustainability by maximizing resource efficiency and product diversity. The dynamic life cycle assessment (dLCA) methodology focuses on assessing emerging biotechnologies such as SSF. This approach performs an iterative assessment that allows for tracking environmental impacts associated with constantly evolving technological development trajectories [86]. The fundamental premise is that as technological innovation advances, the environmental impacts of emerging technologies change substantially. To address the challenges arising from data scarcity, it is crucial to foster systematic collaboration between LCA modelers and experimental teams [82,86]. The role of SSF in transforming waste in biorefineries emphasizes its potential to produce a range of bioproducts, thereby advancing a circular economy. Developing integrated biorefinery models that combine SSF with other processes can enhance environmental and economic sustainability by maximizing resource efficiency and product diversity [90].

In conclusion, SSF presents numerous opportunities for sustainable bioprocessing, including the overcoming regulatory hurdles, enhancing microbial strain performance, and effectively integrating into the circular bioeconomy. These critical areas require continued research and collaborative efforts. The application of SSF to these wastes not only contributes to the production of high-value-added compounds but also promotes sustainability and the circular economy by reducing the environmental impact of agri-food industrial waste.

### 4.7. Regulatory and Market Challenges

The commercialization of products derived from SSF faces regulatory hurdles, especially in sectors like food and pharmaceuticals, where stringent safety and quality standards are enforced. Navigating these regulatory landscapes requires a comprehensive understanding and compliance, which can be resource-intensive [13]. A review of global regulatory frameworks for fermented foods highlights the complexity and variability of regulations across jurisdictions, underscoring the need for harmonization to facilitate market entry [113]. Additionally, market acceptance of SSF-derived products depends on consumer awareness and perception. Efforts to educate consumers about the benefits of SSF, such as sustainability and waste reduction, are crucial for market penetration. A systematic review identified key factors driving consumer perception and preference for fermented food products, including taste, health benefits, and environmental impact, suggesting that targeted communication strategies can enhance acceptance [114].

The 2023 State of the Industry Report on fermentation discusses the importance of developing regulatory frameworks that keep pace with technological advancements to support the commercialization of fermentation-derived products [115]. The U.S. Food and Drug Administration (FDA) focuses on the product, emphasizing its safety rather than manufacturing processes. Products generated by microorganisms enter the market through the “Generally Recognized as Safe” (GRAS) pathway. However, the initiative for new mandatory “bioengineered” labeling requirements has added further complexity [116].

### 4.8. Integration with Other Biotechnological Processes

To maximize its impact and full incorporation into the circular economy, SSF must be integrated with other technologies, such as anaerobic digestion, which leads to improved methane production when using SSF as a pretreatment for municipal solid waste [117]. Another way SSF fits more effectively into circular economy schemes is by obtaining products that function as additives to improve the anaerobic digestion process. An example of this is the production of sophorolipids via SSF of molasses with winterization oil cake. The use of low concentrations of sophorolipids helps increase methane production by up to 41% without significantly altering the microbial community used in anaerobic digestion [14]. SSF can be optimized by integrating it with other biotechnological processes across three areas: pretreatment to improve nutrient availability; the use of additives to enhance microbial activity and maximize metabolite production; and byproduct reuse to promote sustainability. Hybrid systems further maximize production efficiency by leveraging the strengths of these methods. This comprehensive approach is a promising strategy for improving efficiency, sustainability, and profitability in bioproduct production [14,104].

Another example of incorporating SSF into anaerobic digestion processes is the use of its resultant waste, with the particularity of having been obtained with biochar incorporation to avoid high concentrations of compounds such as ammoniacal nitrogen and sodium in cationic form. These affect SSF processes involving microorganisms such as *Pleurotus ostreatus*. Thus, SSF is coupled with the reuse of waste from techniques that, in turn, enable further reuse, thereby maximizing the use of waste in accordance with the principles of the circular economy [118]. Therefore, future research should focus on developing hybrid systems in which SSF is part of a comprehensive scheme for the use of biomass, combining it with other processes to generate multiple value-added products.

## 5. Conclusions and Outlooks

The current and future perspectives of advances in SSF for designing safe and sustainable bio inputs from wastes and byproducts as raw materials highlight the valorization of food and agro-industrial wastes. SSF has emerged as a promising method for converting food and agricultural industrial byproducts (e.g., husks, fruit peels, and sugarcane bagasse) into high-value bioinputs such as biofertilizers, biopesticides, and growth-promoting enzymes. Another current aspect is microbial resource optimization. Advances in microbial engineering have enabled the selection and modification of microorganisms (e.g., fungi and bacteria) that are efficient in degrading complex substrates and synthesizing bioactive compounds under SSF conditions.

A current topic of SSF is the design of enhanced bioprocessing techniques, considering innovations in bioreactor design and process control (e.g., moisture management, aeration, and temperature regulation) that improve the efficiency, scalability, and consistency of SSF processes. From our perspective, one of the most current aspects of SSF is its environmental and economic benefits, as it contributes to waste minimization, reduces greenhouse gas emissions, and offers a cost-effective approach to bioinput production compared to liquid fermentation systems. Among the future perspectives of SSF is its integration into circular bioeconomy models, as SSF can be a cornerstone of circular bioeconomy strategies, where waste streams are fully utilized to produce sustainable bioinputs, thereby closing resource loops. Also, the development of multifunctional bioinputs is an important challenge for SSF. For this reason, further studies and research projects should focus on creating bioinputs with multiple functionalities, such as biostimulants that also act as pest deterrents or improve soil health.

In our experience, the exploration of novel substrates must be a continuous part of the SSF bioprocess. Expanding the range of substrates to include urban organic waste, food waste, and other underutilized residues could unlock new opportunities for sustainable bioinput production. Another challenge associated with some advances is the use of digital and AI-driven optimization, incorporating machine learning and AI tools that can optimize SSF parameters, predict product yields, and enhance scalability for industrial applications. Finally, the implementation of appropriate policy and regulatory support, encouraging sustainable practices through supportive policies, incentives, and regulatory frameworks, will facilitate the adoption of SSF technologies globally. In conclusion, SSF can transform waste and byproducts into valuable bioinputs, align with global sustainability goals, and offer a pathway to eco-friendly agricultural and food industrial practices.

## Figures and Tables

**Table 1:** Microorganisms used in SSF with different substrates to obtain compounds.

Microorganism	Substrate	SSF Conditions	Product/Result Obtained	Reference
*Fungi*				
*Aspergillus awamori*	Orange peels (ORAs) and exhausted sugar beet cossettes (ESBCs).	5 g substrate, 70% moisture, 30 °C, inoculum: 10^7^ spores/g.	High xylanase and exopolygalacturonase enzyme activities.	[53]
*Aspergillus niger*	Crude olive pomace (COP), vine shoots trimming (VTS), brewer’s spent grain (BSG), and exhausted olive pomace (EOP).	10 g substrate, 75% moisture, inoculum: 10^6^ spores/mL.	Increased production of lignocellulolytic enzymes (β-glucosidase, celulase, and xylanase).	[55]
*Trametes versicolor*	Barley husk.	50 g substrate, 59% moisture, 27 °C, inoculum: five mycelial plugs.	High production of laccase.	[40]
*Pleurotus ostreatus*	Apple bagasse.	10 g substrate, 80% moisture, 25 °C, inoculum: 1 cm diameter mycelium-agar circle (3 mg biomass).	Antioxidant activity, total triterpenes, phenolic compounds, and flavonoids.	[41]
*Aspergillus niger*	Granadilla seeds.	5 g substrate, 5.5% moisture, 30 °C, inoculum: 1.6–3.6 × 10^7^ spores/g.	High phenolic content (4713.3 GAE/100 g flour) and total flavonoid (1910.4 mg quercetin/100 g flour).	[56]
*Aspergillus niger*	Moringa leaves.	5 g substrate, 50–70% moisture, 30 °C, inoculum 1.0–4.0 × 10^7^/g of solid.	Increased total phenolics (136.4%) and flavonoids (783.1%). High FRAP antioxidant activity (277.2 μmol Trolox/g).	[57]
*Trichoderma reesei*	Sugarcane bagasse and Wheat bran	10 g substrate, 40% moisture, 30 °C, inoculum: seed culture 6.7 g.	Ethanol production (14.1%).	[58]
*Yeast*				
*Saccharomyces cerevisiae*	Coffee pulp (*Coffea arabica*)	400 g substrate, 85% moisture, 28 °C, inoculum: 10^7^ CFU g^−1^.	Extract containing 300 to 400% more chlorogenic acids than its initial concentration.	[59]
*Saccharomyces cerevisiae*	Rambutan Peel.	1.5 g substrate, 60% moisture, 30 °C, inoculum: 1.5 × 10^7^ cells/g.	High ellagic acid accumulation (458.37 ± 44.6 mg/g).	[42]
*Kluyveromyces marxianus*	Beer residue.	5 g substrate, 75% moisture, 37.5 °C, inoculum: 10^8^ cells/mL.	High inulinase activity of 239.38 U/g.	[60]
*Yarrowia lipolytica*	Soybean hulls.	0.5 g substrate, 55% moisture, 28 °C, inoculum: 0.71 mg/g.	Increased lipase production (1.58 kU/L).	[61]
*Bacteria*				
*Pseudomonas aeruginosa*	Sugar cane bagasse and sunflower seed meal.	10 g substrate, 30 °C, inoculum: 2 × 10^9^ CFU/mL.	67% improvement in rhamnolipid levels, higher than that obtained by SSF.	[62]
*Streptomyces* sp.	Wheat straw.	10 g substrate, 85% moisture, 28 °C, inoculum: 10^7^ CFU/mL.	High xylanase and mannanase enzyme activities.	[63]
*Bacillus subtilis*	Soybean meal.	1:1.25 substrate radio, 55% moisture, 45 °C, inoculum 25% (*v*/*w*).	High peptide yield (220.93 mg/g).	[64]

## Data Availability

No new data were created or analyzed in this study. Data sharing is not applicable to this article.

## Abbreviations

The following abbreviations are used in this manuscript:

SSF	Solid-state fermentation
SmF	Submerged fermentation
MSW	Municipal solid waste
LCAs	Life cycle assessments
dLCA	Dynamic life cycle assessment
GMOs	Genetically modified organisms
LCA	Life cycle analysis
CEI	Circular economy index
MFA	Material flow analysis
AI	Artificial intelligence
